# Processing maize flour and corn meal food products

**DOI:** 10.1111/nyas.12299

**Published:** 2013-12-11

**Authors:** Jeffrey A Gwirtz, Maria Nieves Garcia-Casal

**Affiliations:** 1 Department of Grain Science and Industry, Kansas State UniversityManhattan, Kansas; 2 Laboratory of Pathophysiology, Experimental Medicine Center, Venezuelan Institute for Scientific ResearchCaracas, Venezuela

**Keywords:** maize, corn, processing methods, flour, food fortification

## Abstract

Corn is the cereal with the highest production worldwide and is used for human consumption, livestock feed, and fuel. Various food technologies are currently used for processing industrially produced maize flours and corn meals in different parts of the world to obtain precooked refined maize flour, dehydrated nixtamalized flour, fermented maize flours, and other maize products. These products have different intrinsic vitamin and mineral contents, and their processing follows different pathways from raw grain to the consumer final product, which entail changes in nutrient composition. Dry maize mechanical processing creates whole or fractionated products, separated by anatomical features such as bran, germ, and endosperm. Wet maize processing separates by chemical compound classification such as starch and protein. Various industrial processes, including whole grain, dry milling fractionation, and nixtamalization, are described. Vitamin and mineral losses during processing are identified and the nutritional impacts outlined. Also discussed are the vitamin and mineral contents of corn.

## Introduction

Maize is a domesticated grass that originated approximately 7000 years ago in what is now Mexico. It is also referred to as corn, and both words are used as synonyms in this review, depending on the source of data or references consulted. Maize was spread across the world shortly after the European discovery of the Americas. Regardless of origin, corn has proven to be one of the most adaptable crops. Its evolution apparently occurred mainly under domestication and resulted in biotypes with adaptation ranging from the tropics to the north temperate zone, from sea level to 12,000 feet altitude, and growing periods (planting to maturity) extending from 6 weeks to 13 months.^1^,^2^ Currently, the United States, Brazil, Mexico, Argentina, India, France, Indonesia, South Africa, and Italy produce 79% of the world's maize production.^3^ Between 1990 and 2011, the number of millions of maize hectares harvested ranged from 129.1 to 163.9. During the same period the production of maize in metric tons per hectare increased from 3.7 to 5.1, and total maize production increased from 482.0 to 832.5 million metric tons. Worldwide, 60–70% of maize production is used domestically as livestock feed, and the remaining 30–40% is used for production of items for human consumption.^4^

Corn is the main cereal grain as measured by production but ranks third as a staple food, after wheat and rice. The reasons for this fact are varied, but some of them are related to cultural or social preferences and also because in some countries, corn is cultivated as livestock feed. More recently, the use of corn as a biofuel has generated great concern about rises in the market price of corn for consumption, the need to increase cultivable areas, as well as water quality and other ecological damages. Some predictive models project that large-scale corn ethanol production could lead to decreases in food exports, higher prices, and a greater global deforestation.^5^,^6^

## Maize kernel anatomy

**Figure 1:**
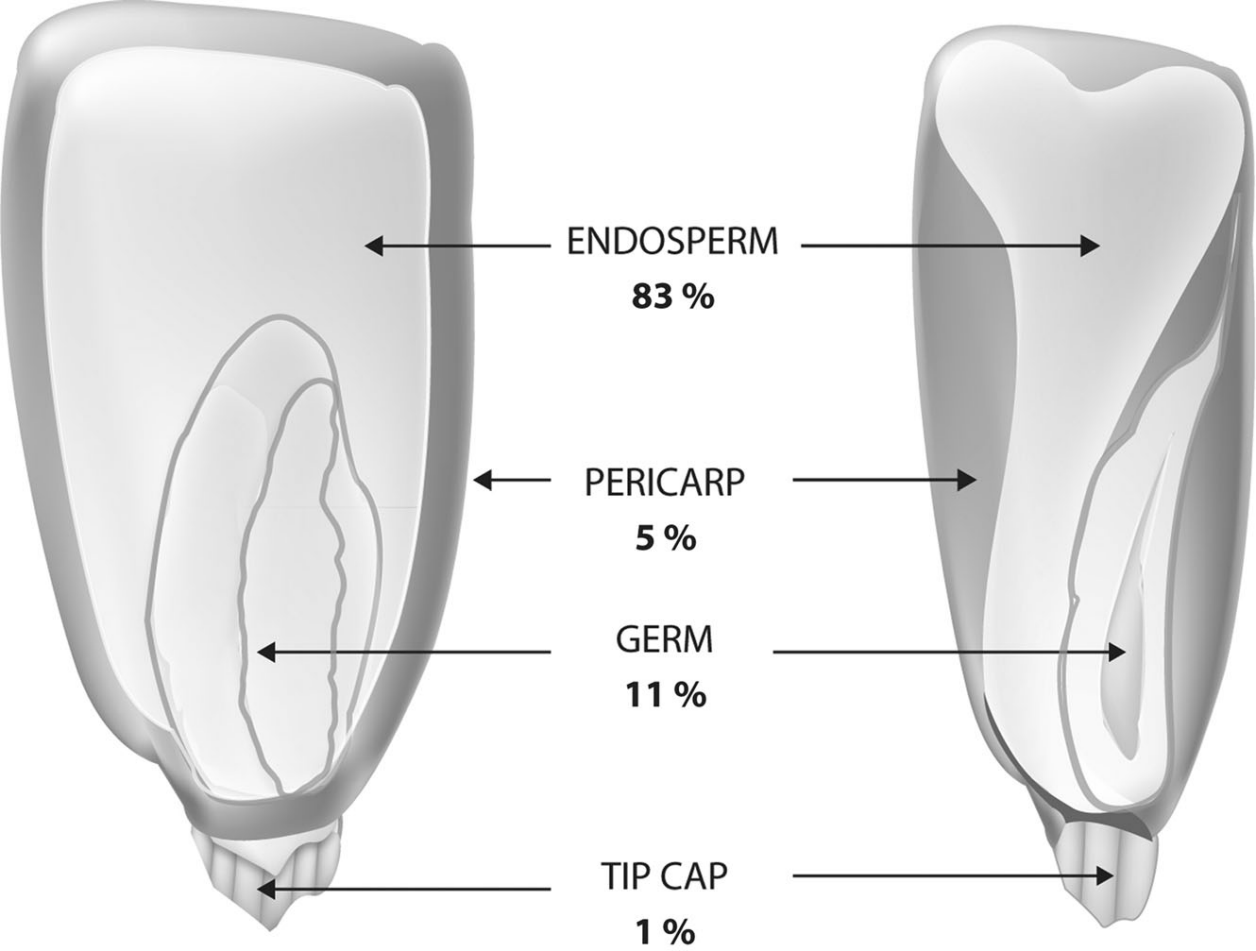
Components of the corn kernel.

The maize kernel is composed of four primary structures from a processing perspective. They are endosperm, germ, pericarp, and tip cap, making up 83%, 11%, 5%, and 1% of the maize kernel, respectively (Fig. 1). The endosperm is primarily starch surrounded by a protein matrix. Two main types of starch include hard or vitreous, and soft or opaque. Vitreous endosperm is negatively related to starch degradability and *in vivo* starch digestibility in ruminants.^7^,^8^

The germ or embryo of the maize kernel is high in fat (33.3%) in addition to enzymes and nutrients for new maize plant growth and development. The germ also contains vitamins from B complex and antioxidants such as vitamin E. Maize germ oil is particularly high in polyunsaturated fatty acids (54.7%), which are subject to oxidative and other forms of rancidity resulting in off or objectionable flavors from full-fat maize products. Pericarp is a high-fiber (8.8% crude) semipermeable barrier surrounding the endosperm and germ, covering all but the tip cap. The tip cap is the structure through which all moisture and nutrients pass through during development and kernel drydown. The black or hilar layer on the tip cap acts as a seal.^9^ The term *bran* is also used to refer to the fiber-rich outer layer (pericarp) that contains B vitamins and minerals and the tip cap.

Corn variations may be artificially defined according to kernel type as follows: dent, flint, waxy, flour, sweet, pop, Indian, and pod corn. Except for pod corn, these divisions are based on the quality, quantity, and pattern of endosperm composition, which defines the size of the kernel, and are not indicative of natural relationships. Endosperm composition may be changed by a single gene difference, as in the case of floury (fl) versus flint (FI), sugary (su) versus starchy (Su), waxy (wx) versus nonwaxy (Wx), and other single recessive gene modifiers that have been used in breeding special-purpose types of corn.^1^,^10^

## Maize kernel composition

Tables1 and 2 provide the vitamin and mineral analysis of corn, crude bran, and cornstarch as available from the U.S. Department of Agriculture Nutritional Data Base.^11^ As can be observed, the corn bran is a significant contributor to maize vitamin and mineral content. The wet milling of maize separates much of its nutrient content away from the starch component.

**Table 1:** Vitamin content of whole kernel, crude bran, and corn starch of yellow corn

	Unit/	Corn,	Corn,	Corn,
Vitamin	100 g	whole	bran	starch
Thiamin	mg	0.39	0.01	0
Riboflavin	mg	0.20	0.10	0
Niacin	mg	3.63	2.74	0
Pantothenic acid	mg	0.42	0.64	0
Vitamin B6	mg	0.62	0.15	0
Folate	μg	19.00	4.00	0
Choline	μg		18.10	0.40

Note: Data from U.S. Department of Agriculture.^11^

**Table 2:** Mineral content of whole kernel, crude bran, and corn starch of yellow corn

	Unit/	Corn,	Corn,	Corn,
Mineral	100 g	whole	bran	starch
Calcium, Ca	mg	7.00	42.00	2.00
Iron, Fe	mg	2.71	2.79	0.47
Magnesium, Mg	mg	127.00	64.00	3.00
Phosphorus, P	mg	210.00	72.00	13.00
Potassium, K	mg	287.00	44.00	3.00
Sodium, Na	mg	35.00	7.00	9.00
Zinc, Zn	mg	2.21	1.56	0.06
Copper, Cu	mg	0.31	0.25	0.05
Manganese, Mn	mg	0.49	0.14	0.05
Selenium, Se	μg	15.50	16.5	2.80

Note: Data from U.S. Department of Agriculture.^11^

In addition to chemical composition, physical characteristics of maize in the commercial market place influence the value of the grain or the final product. Often, countries will have grading standards for maize entering the supply chain to assist buyers and sellers assessing maize value. Test weight, moisture content, foreign material, and damage are among typical measures of maize quality and value.^12^

## Maize pathways to the consumer

**Figure 2:**
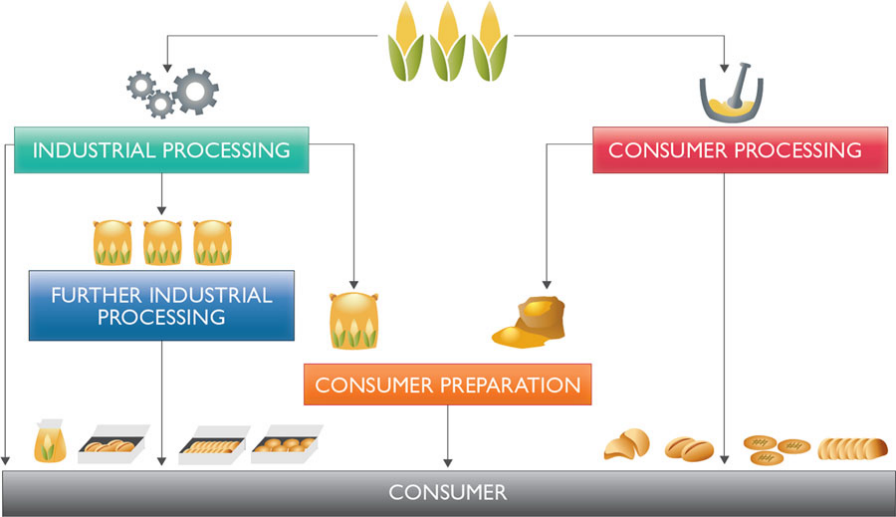
Pathways of maize from field to consumer.

Maize food products can be processed at home on a small local scale as well as on a larger industrial scale, transforming the raw material into food products (Fig. 2). Some of the products are more suitable for commercial trade because they require further processing or provide convenience and extended shelf life, while other products should be consumed immediately after production. For example, degerminated corn grits, meal, or flour has an extended shelf life and can be moved and traded easily. The product itself has to be further processed, including some degree of cooking, in order for it to be palatable as a food product.

Nixtamalized maize, when prepared in the household or by a small-scale processor, is typically used to form ready-to-eat finished masa products with a limited shelf life. On an industrial scale, nixtamalized maize flour may be processed and sold as a shelf-stable product that can be prepared for consumption in the home, reducing meal preparation time and providing convenience. Industrial processors strive to offer maize products that replicate the ones consumed in the target market region. Identifying the market share of industrially produced maize product consumed in the market place is essential in evaluating the potential for implementing a successful maize product fortification program. The influence of a small-scale maize processing industry with local impact may also play a significant role in some target populations, especially in some poor countries.

Maize color is important to specific consumer groups in Central and South America as well as in Africa, and white maize is preferred for food consumption. In some areas of the world, such as North America, the desired color depends on the region or the food use. For example, in the eastern United States, cornmeal, grits, and homily are white, while in the northern part of the country, maize meal, and maize products used for breakfast cereals and snack foods are expected to be manufactured with yellow maize.

Maize meal or flour composition can also be driven by regional preferences, with some preferring whole ground maize rather than degerminated or partially degerminated maize products. The nutrient composition, including vitamins, minerals, and antinutrient factors, are influenced by local product preferences, which include not only the way the corn product is consumed, but also what other food items or additives are part of a complete meal. Local and regional standards for both the form and composition are to be evaluated as part of planning a maize flour or cornmeal fortification program.

Maize products and processing methods are as diverse as the maize crop itself. Processing of maize at the household or local industrial level may be accomplished with wooden mortar and pestles, stone metates, and manos (stones).^13^ Particle reduction in germ and/or fiber content may be accomplished with screening. Poor product stability, especially due to fat content, results in the need for frequent processing of maize at the household and small-scale industry level. Additionally, the number of nutrients removed or altered through home or small-scale industry processing may vary widely. As a result, fortification of maize processed at home or in small-scale industrial mills may not be practical. On the other hand, in some countries, local or household production and/or processing could account for the majority of maize consumption. In those countries, central or nationwide maize fortification programs would not be a solution for improving micronutrient intake.

## Industrial maize processes

There are two basic categories of industrial processing employed for transforming maize into products for human consumption. They are known as dry and wet milling. In the wet milling process, maize is separated into relatively pure chemical compound classes of starch, protein, oil, and fiber. The products and coproducts obtained from wet maize milling are not typically directly used by the consumer and often require further industrial processing before consumption. The products of wet maize milling are not typically produced on a small scale commercially or in the home. The primary product, starch, can be processed into a variety of starch products or further refined into a variety of sweeteners sold in liquid and dry forms. Wet milling of maize will not be further addressed in this article.

Industrial dry milling includes particle size reduction of clean whole maize with or without screening separation, retaining all or some of the original maize germ and fiber.^14^ Because of the high-fat content, these whole or partially degerminated maize products are not particularly shelf stable. Degermination of maize involves mechanical separation and processing, resulting in dry shelf-stable products with a majority of both germ and fiber removed. Much of the particle size reduction and separation is accomplished with equipment similar to that employed in wheat flour milling, including hammer mills, stone mills, roller mills, screeners, sifters, specific gravity separators, and aspirators. Specialized equipment, such as degerminators and de-hullers or peelers, may be employed in maize processing.

Generally, whole, partially degerminated, and degerminated maize products require additional processing before consumption. These processing steps may be accomplished in a large-scale industrial setting, small-scale local processor, or in the home. These secondary processes may include addition of other ingredients along with thermal processing, including boiling, drying, frying, or baking, all of which can affect the nutritional attributes of the finished product.

A second type of industrial dry maize processing is alkali processing or nixtamalization in which whole maize is cooked with an excess of water treated with calcium oxide.^13^ The maize kernel may be ground whole, fractionated, or have other corn components added. Unlike wheat flour milling, processing equipment for alkali-treated corn is specialized to handle the moisture, chemicals, and heat required for wet processing. Conventional dry bulk material handling and processing equipment is employed with raw maize and dry finished product. The resulting intermediate product may be dried for commercial sales of further processed consumer food product. In North America and Mexico, dry alkali-processed maize flour is known as masa flour, a name that is not used in Spanish-speaking countries of Central and South America or non-Spanish speaking countries of Africa. These products are referred herein as alkali processed. The alkali process improves flavor, starch gelatinization, and water uptake. The process partially removes some of the germ and most of the pericarp, but the amount varies. In some cases, pericarp may be added into the process for visual product enhancement. The heating in the process causes loss of thiamine, riboflavin, niacin, fat, and fiber. As might be expected the calcium content increases owing to the alkali processing.

In the nixtamalization process, there are several stages. First, dried maize is soaked in a solution of water with lime, often with ashes mixed in. The grain is then cooked, steeped, drained, and rinsed multiple times. The grain is then ground to make a wet dough from which tortillas are formed or allowed to dry into flour. Currently, there is an important diminution in production of homemade tortillas because they are now prepared from commercial instantaneous flour or bought as packaged tortillas.^15^,^16^ Nixtamalized maize has several benefits compared to unprocessed grains: they are more easily ground and have a higher nutritional value (increased bioavailability of niacin, improved protein quality, increased calcium) and reduced mycotoxins content.^17^

A staple maize product in South America, particularly in Venezuela and Colombia, is arepa, which is a fried or baked bread prepared from precooked refined corn flour.

Traditionally, arepas are made by dehulling and degerming previously soaked whole kernels by manually grinding maize kernels in a pilon, a wooden mortar. The bran and germ are removed by repeatedly rinsing the mixture containing the endosperm with water. This fraction is then cooked and milled to prepare a dough that will be shaped and cooked (baked or fried) to obtain the arepa.^18^ The traditional process for preparing homemade arepas involves soaking, cooking, cooling, draining, grinding, and forming a dough piece for additional grilling or baking. The process takes 18 or more hours to complete in the home.

The traditional method has been modified with the introduction of precooked maize flour.^19^ This process includes conditioning, cooking, flaking, drying, grinding, and sifting to produce dry instant precooked refined arepa flour. The flour can be transported and stored easily until used in the home. Preparation is reduced to less than an hour, making it more convenient for consumers.^20^–^23^ Although arepa is prepared from flour that is 100% corn, there are commercial presentations of mixtures of corn with rice, corn with wheat, and corn with oat and wheat bran.

Fermented maize products, such as ogi, are prepared by soaking the maize kernel for 1–3 days until soft. It is then grinded with a stone and dehulled and degermed by repeated washes with water. The filtered endosperm is fermented for 2 or 3 days, producing a slurry that becomes the ogi porridge when boiled. Fermented products that are similar but prepared with different maize varieties or minor preparation changes include uji in Kenya, kenkey, banku, ogi, and koko from Ghana and Nigeria.^24^,^25^ Nixtamal is another fermented maize product, prepared from dehulled kernels ground to a coarse dough and wrapped in banana leaves to ferment for 2 or 3 days.^20^

**Figure 3:**
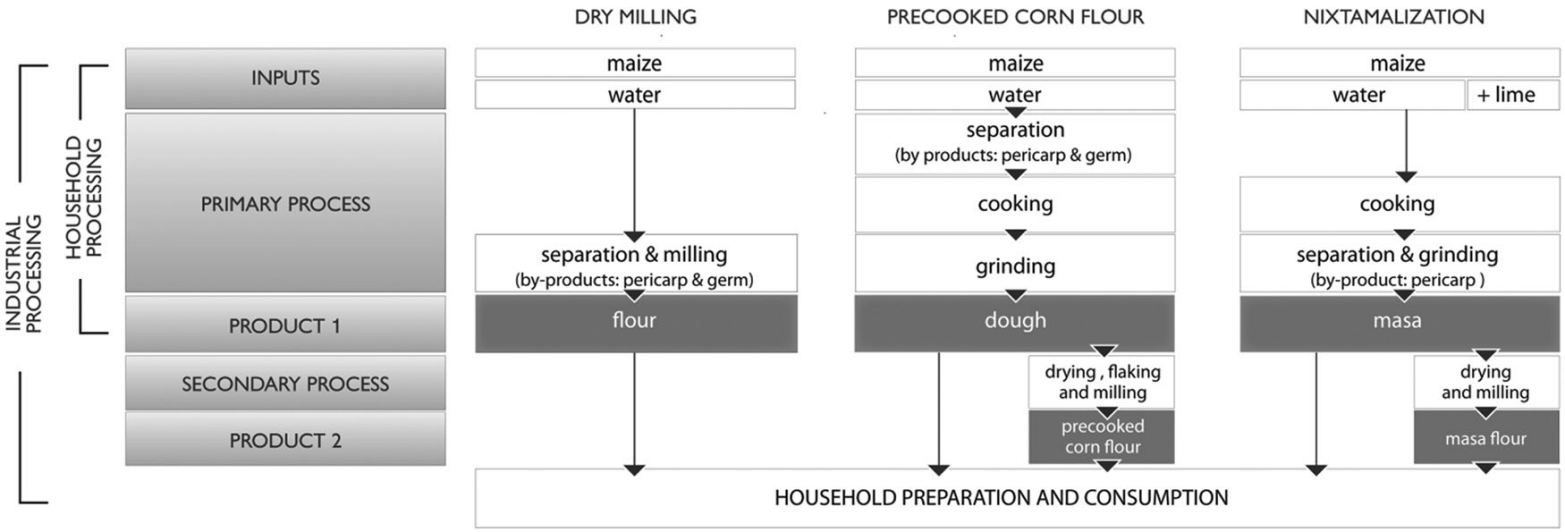
Schematics of dry-milling maize processing.

Figure 3 provides a schematic of three of the maize dry-milling processes: whole or refined, nixtamalized, and precooked corn flours. There is a common, similar initial process between household and industrial preparation of nixtamalized and precooked corn flours that is taken from the traditional way of household preparation. At the point of product 1, the masa or dough is obtained and final products could be prepared. Industrial processing from this point produces the commercial flour that only needs added water and to be cooked at the household level, to obtain the traditional product without repeating all the processing on a daily or regular basis.

Separation of maize constituents (e.g., dehulling or degerming) varies depending on regional customs and consumer preference. These differences affect the vitamin and mineral content of the finished product from primary processing and should be taken into consideration when developing a maize fortification strategy. Yield, fat, and fiber content of the maize product from the primary processes will be directly proportional to nutrient content. Particle size will also be important to the fortification strategy as presented later in the article. Fortification of products from the secondary processing of maize becomes widely varied and inherently more difficult to manage.

The products derived from dry milling are numerous, with their variety depending to a large extent on particle size. In Africa, ground maize is cooked into a paste accompanied by a thick low-alcoholic beer. This maize paste could be fried or baked, depending on the region of Africa. Many Africans depend on some variation of this mush, which is made with water and ground maize. It can also be eaten as a porridge or a dumpling, depending on the thickness of the batter and the cooking method.^26^ In Kenya, they prepare uji, a porridge of maize flour cooked in water and sweetened with sugar.^27^

Other maize preparations include humitas prepared from precooked maize flour, mote made from cooked maize and cheese, pupusas made from lime-treated maize and cheese, and patasca, which is like a lime-treated maize kernel.^20^ Table 3 shows a variety of maize products of global interest.^13^

**Table 3:** Various maize products consumed globally

Bread	
Flat, unleavened, unfermented	Tortilla, arepa
Fermented and/or leavened	Pancakes, cornbread, hoe cake, blintzes
Porridges	Atole, ogi, kenkei, ugali, ugi, edo, pap,
Fermented, unfermented	maizena, posho, asidah
Steamed products	Tamales, couscous, rice-like products, Chinese breads, dumplings, chengu
Beverages	
Alcoholic	Koda, chicha, kafir beer, maize beer
Nonalcoholic	Mahewu, magou, chicha dulce
Snacks	Empanadas, chips, tostadas, popped corn, fritters

Note: From Rooney and Serna-Saldavar^13^

Attempts have been made to classify and define products of maize processing; however, there is not a globally recognized terminology for dry-milled maize products.^9^ Table 4 identifies the commonly accepted terms used according to ranges of particle size for maize products. The fat values are for degerminated maize products.^28^ Some have subdivided the definition of maize meal into smaller size categories to include coarse meal (1190–730 μm), medium meal (730–420 μm), and fine meal or cones (420–212 μm).^29^

**Table 4:** Degerminated maize products defined by particle size and fat content

	Particle size		
	Less than (μ)	Greater than (μ)	Fat (%)
Grits	1400	600	0.8
Meal	600	300	1.8
Fine meal	300	212	2.5
Flour	212		2.7

Note: Data from Baltenspreger.^18^

The U.S. Code of Federal Regulations, Title 21, provides standards for various maize products, such as white corn flour (137.211), yellow corn flour (137.215), white corn meal (137.250), enriched cornmeal (137.260), degerminated white cornmeal (137.265), and self-rising white cornmeal (137.270).^30^ It is important to note that publicly available standards do not always identify end-use properties. It is possible that some aspects of a government-provided standard for a product may be less restrictive than the commercial standard of the customer. In some countries, the government and consumer standard is one and the same regulation.^31^,^32^

Many products of the industrial dry maize–milling processes may also be produced locally on a small scale as well as in the home. High-moisture wet products from local small-scale producers have limited shelf life and must be used in a short period. However, the products are still interesting for users because they reduce preparation time for the homemaker. When produced in the home the process is generally carried out to a food product level ready for consumption.

## Fortification of maize products

Fortification of maize flour or cornmeal with iron is a cost-effective, food-based approach that should be regarded as part of a broader, integrated initiative to prevent micronutrient malnutrition, complementing other efforts to improve micronutrient status. Other initiatives include supplementation, change of food habits, food-to-food fortification, point-of-use fortification, promotion of increased consumption and/or production of food, improvement of health and sanitary conditions, biofortification, genetically modified foods, and nanotechnologies.

Identification of maize consumption volume as well as product type and significant maize product production capacity at the industrial levels is required for a successful maize fortification program. Maize flour, masa, or dry maize products from the primary processing identified in Figure 3 with less than 13% moisture content are stable and may be fortified with a powdered premix composed of appropriate vitamins and minerals that could be similar to those used in wheat flour fortification programs^33^,^34^ but are always based on the needs of a particular population, country, or region and not on flour capabilities of accepting a particular premix. The fine maize products (100% less than 600 μm) are suitable for the addition of powdered premix and will not be subject to significantly meaningful segregation. The selection of the adequate micronutrient mixture is key for a program to be successful. It is important to select a mixture of micronutrients, especially regarding iron, that is well absorbed and at the same time does not change the organoleptic characteristics of the fortified maize flour, cornmeal, or the meals that contain the fortified flour. A Flour Miller's Toolkit for flour fortification has recently been updated to provide practical insights for flour fortification within the context of a grain milling or processing facility.^35^

Table 5 provides a proximate vitamin and mineral analysis for white maize, whole-grain maize flour, degerminated meal (unenriched and enriched), alkali-processed maize or masa flour (unenriched and enriched), and precooked corn flour (unenriched and enriched).^11^,^36^ Degerminated maize products are clearly lower in fat, fiber, and ash content when compared to whole maize or whole maize flour. The influence of alkali processing on the calcium level of the finished product is readily apparent given the higher values of calcium reported.

**Table 5:** Proximal analysis of vitamin and mineral content of white corn, flour, meal, and alkali-processed masa, unenriched and enriched

				Corn flour,	Cornmeal,	Cornmeal,	Corn flour,	Corn flour,	Precooked	Precooked
			Corn flour,	degermed,	degermed,	degermed,	masa,	masa,	corn flour,	corn flour,
	Unit/100g	Corn	whole grain	unenriched	unenriched	enriched	unenriched	enriched	unenriched	enriched
Water	g	10.4	10.9		11.2	11.2	9.0	9.0	11.2	11.2
Energy (Kcal)	Kcal	365	361		370	370	365	365	354	354
Energy (KJ)	KJ	1527	1510		1547	1547	1528	1528		
Protein (*N*×6.25)	g	9.4	6.9		7.1	7.1	9.3	9.3	7.2	7.2
Total lipids	g	4.7	3.9		1.8	1.8	3.9	3.9	1.1	1.1
Ash	g	1.2	1.5		0.5	0.5	1.5	1.5	0.3	0.3
Carbohydrates	g	74.3	76.9		79.5	79.5	76.3	76.3	80.2	80.2
Total fiber	g		7.3		3.9	3.9	6.4	6.4	2.5	2.5
Sugars, total	g		0.6		1.6	1.6	1.6	1.6		
Starch	g				73.3	73.3	66	66		
Thiamin	mg	0.39	0.25	0.07	0.14	0.55	0.22	1.48	0.06	0.31
Riboflavin	mg	0.20	0.08	0.06	0.05	0.38	0.1	0.81	0.05	0.25
Niacin	mg	3.63	1.9	2.66	1	4.97	1.63	9.93	0.6	5.1
Pantothenic acid	mg	0.42	0.66	0.05	0.24	0.24	0.19	0.19		
Vitamin B6	mg	0.62	0.37	0.1	0.18	0.18	0.48	0.48		
Folate, total	μg		25	48	30	209	2.9	209		
Folic acid	μg		0	0	0	180	0	180		
Folate, food	μg		25	48	30	30	2.9	2.9		
Folate, FDE	μg		25	48	30	335	2.9	335		
Choline	mg		21.6		8.6	8.6	8.6	8.6		
Calcium, Ca	mg	7	7	2	3	3	136	136	12	12
Iron, Fe	mg	2.7	2.4	0.9	1.1	4.4	1.5	7.5	0.9	5.0
Magnesium, Mg	mg	127	93	18	32	32	93	93		
Phosphorus, P	mg	210	272	60	99	99	214	214	64	64
Potassium, K	mg	287	315	90	142	142	263	263		
Sodium, Na	mg	35	5	1	7	7	5	5		
Zinc, Zn	mg	2.2	1.7	0.4	0.7	0.7	1.8	1.8		
Copper, Cu	mg	0.3	0.2	0.1	0.1	0.1	0.2	0.2		
Manganese, Mn	mg	0.5	0.5	0.06	0.2	0.2	0.4	0.4		
Selenium, Se	μg	15	15.4	8	10.5	10.5	14	14		
Vitamin A	RE									270

Note: Data from U.S. Department of Agriculture^11^ and the Flour Fortification Initiative.^35^

A sustainable fortification program consists of numerous components and actors. In each of these steps, there are many details to control and possible difficulties and barriers to overcome. The components include the preliminary assessment of nutrient deficiencies, the development of fortification standards and legislation, the acquisition of equipment by industrials, communication strategies and social marketing activities, food safety, quality assurance and control systems, and the assessment of the impact of the fortification program on health.

The size, capacity, and number of maize processing facilities as well as process control should be considered in selecting the threshold for maize product fortification intervention. Attempts to fortify maize products at a small local level may not be cost-effective and risk having a negative impact on fortification program perception if not properly executed.^37^ It is beyond the goal of this article to make the maize processing scale cut-off for a fortification program, as well as to discuss bioavailability of nutrients remaining after different processes of corn milling.

## Conclusion

Maize is a significant food source for much of the world's population and represents a vehicle for vitamin and mineral deficiency intervention. There are several industrial processes that generate a wide variety of maize products to fulfill consumers' habits and preferences. Many products of the industrial dry maize–milling processes may also be produced locally on a small scale as well as in the home. The materials, processes, and equipment are readily available, but it is important to consider that the number of nutrients removed or altered through home or small-scale industry processing may vary widely. Proper assessment of population needs and understanding of industrial capability, products, and losses are needed to determine the viability of maize product fortification.
